# Molecular characterization of a novel partitivirus isolated from *Rhizoctonia solani*

**DOI:** 10.3389/fmicb.2022.978075

**Published:** 2022-09-20

**Authors:** Xiangru Chen, Zhaoyao Yu, Yujia Sun, Meipeng Yang, Ning Jiang

**Affiliations:** ^1^Key Laboratory of Agricultural Microbiology, College of Agriculture, Guizhou University, Guiyang, China; ^2^Agronomic Research Center, Yunnan Academy of Tobacco Agricultural Sciences, Kunming, China

**Keywords:** dsRNA (double-stranded RNA), mycovirus, *Partitiviridae*, *Rhizoctonia solani*, tobacco target spot disease

## Abstract

*Rhizoctonia solani* is a widely distributed plant pathogen that can damage many crops. Here, we identified a novel mycovirus tentatively named Rhizoctonia solani partitivirus 433 (RsPV433) from an *R. solani* (AG-3) strain which caused tobacco target spot disease on flue-cured tobacco. RsPV433 was consisted of two dsRNA segments with lengths of 2450 and 2273 bp, which encoded an RNA-dependent RNA polymerase and a coat protein, respectively. BLASTP results of RsPV433 showed that the closest relative of RsPV433 was Sarcosphaera coronaria partitivirus (QLC36830.1), with an identity of 60.85% on the RdRp amino sequence. Phylogenetic analysis indicated that RsPV433 belonged to the *Betapartitivirus* genus in the *Partitiviridae* family. The virus transmission experiment revealed that RsPV433 can be transmitted horizontally. We further tested the biological effect of RsPV433 on *R. solani* strains and found that the RsPV433-infected *R. solani* strain grew slower than the RsPV433-free strain on the PDA medium and RsPV433 seemed to have no obvious impact on the lesion inducing ability of *R. solani*.

## Introduction

*Rhizoctonia solani* is a common soilborne pathogen. *R. solani* consists of 14 subgroups (AG1–AG14), based on the hyphal anastomosis reaction between *R. solani* strains (García et al., 2006; Yang et al., 2015). This pathogen can cause some symptoms, such as root rot, stem canker, sheath blight, or foliage blight on different host plants, and often lead to severe economic damage (Gonzalez et al., 2011; MatRazali et al., 2021). Shew (1991) reported the target spot disease on flue-cured tobacco for the first time and identified that this disease is caused by *R. solani* (AG-3) (Shew, 1991; Gonzalez et al., 2011), however, AG-2.1 and AG-6 were also be reported to be able to cause tobacco target spot disease (Mercado Cárdenas et al., 2012; Sun et al., 2022). The occurrence of tobacco target spot disease in China has been reported since 2012 and it has become a major disease in the production process of flue-cured tobacco in China (Wu et al., 2012; Xu et al., 2018; Sun et al., 2022).

Mycoviruses are microbes that use fungi as their hosts. Most known mycoviruses cryptically infect their hosts, but some mycoviruses can greatly affect their host, either by hypovirulence or hypervirulence. For example, the Sclerotinia sclerotiorum hypovirulence–associated DNA virus 1 can convert its host, *Sclerotinia sclerotiorum*, from a typical necrotrophic pathogen into a beneficial endophytic fungus (Zhang et al., 2020); infection with Nigrospora non-segmented RNA virus 1 led to hypovirulence of *Nigrospora oryzae* on cotton leaves (Wang et al., 2022b); the Diaporthe pseudophoenicicola chrysovirus 1 can confer hypovirulence to the fungal host of *Diaporthe pseudophoenicicola* (Xu et al., 2022); and the virulence of *Leptosphaeria biglobosa* can be increased by the Leptosphaeria biglobosa quadrivirus 1 (Shah et al., 2020). The role of mycoviruses in the pathogenesis of fungi was well-discussed in previous papers (Abdoulaye et al., 2019; Bian et al., 2020; Kotta-Loizou, 2021). Mycoviruses have been divided into 22 taxa by the International Committee on Taxonomy of Viruses (ICTV) https://talk.ictvonline.org/taxonomy/ (Ghabrial and Suzuki, 2009; Kondo et al., 2013, 2022; Ghabrial et al., 2015; Hillman et al., 2017; Ruiz-Padilla et al., 2021; Myers and James, 2022). *Partitiviridae* is a branch of these 22 taxa, all the viruses in this family are spherical with a size of 34–42 nm and have double-straned RNA (dsRNA) genomes (Nibert et al., 2014). Viruses of this family are divided into five genera, namely, *Alphapartitivirus, Betapartitivirus, Gammapartitivirus, Deltapartitivirus*, and *Cryspovirus* (Nibert et al., 2009; Vainio et al., 2018; Petrzik, 2019). Dozens of partitiviruses have been identified from many types of organisms, such as fungi, nematode, plants, and protists (Shiba et al., 2018; Kamitani et al., 2020; Tang et al., 2021; Wang et al., 2022a). There are many tentative species in this family, such as Botryosphaeria dothidea partitivirus 2 that identified recently (Wang et al., 2022c); Nigrospora sphaerica partitivirus 1 virus infecting *Nigrospora sphaerica* (Zhong et al., 2021); and Rhizoctonia fumigata partitivirus 1, isolated from *Rhizoctonia fumigata* AG-Ba strain C-314 Baishi (Li et al., 2022).

*Rhizoctonia solani* is a host of mycoviruses, so far, there are more than 100 viruses have been found in *R. solani* (Abdoulaye et al., 2019). In this study, we discovered a novel partitivirus tentatively named Rhizoctonia solani prtitivirus 433 (RsPV433), which was isolated from a TS23 strain of *R. solani* that caused tobacco target spot disease on flue-cured tobacco in Yunnan province.

## Materials and methods

### Mediums, fungal strains and plant materials

Potato dextrose agar (PDA) medium (Solarbio, Beijing, China) is the medium we used in most of our experiments. The V8 juice agar (V8A) medium [15% clarified V8 juice (Campbell Soup Co., NJ, USA) with 2.5 g/L CaCO_3_ and 2% agar] is prepared follow the instructions described previously (Feng et al., 2022).

TS10 and TS23 are *R. solani* strains that were isolated by Ning Jiang from tobacco target spot leaf samples collected in Yunnan province. According to the former study, we knew that tobacco target spot leaf disease is caused by *R. solani* (Shew, 1991; Gonzalez et al., 2011; Mercado Cárdenas et al., 2012; Sun et al., 2022). To get more information about these strains, we further amplified the ITS region of TS10 and TS23 by primer sets ITS1/ITS4 (White et al., 1990). NCBI blast results of the amplified fragments of TS10 and TS23, combined with the field symptom and the morphological observation results, we speculated that these two strains belong to the AG-3 group of *R. solani*. TS10-23 is the offspring of the recipient TS10 strain in the horizontal transmission experiment, it is an *R. solani* TS10 strain infected by RsPV433. These strains and their subcultures were incubated on PDA plates at 25°C and stored at 4°C on PDA slants.

*Nicotiana tobaccum* and *N. bethamiana* were grown in pots with garden soil in a growth chamber under a 10:14 h (L:D) photoperiod at 25°C.

### dsRNA extraction

The *R. solani* strains were cultured on cellophane membranes overlaid on the surfaces of PDA plates at 25°C for 4 days in darkness (Yang et al., 2015). Mycelia of cultured strains were harvested and ground into a fine powder in liquid nitrogen. dsRNAs were extracted using CF-11 cellulose (Sigma-Aldrich, Dorset, England) following previously described procedures (Morris and Dodds, 1979; Zhai et al., 2016). All the extracted dsRNAs were digested with S1 nuclease and DNase I (Takara, Dalian, China) to eliminate DNA and single-stranded RNA (ssRNA) contamination and kept at −80°C.

### cDNA cloning and sequence analysis

Cloning and sequencing of the dsRNAs were conducted as previously described (Xie et al., 2011; Wang et al., 2013; Li et al., 2015). First, the purified dsRNAs were reverse-transcribed by a tagged random primer dN6, and then these random cDNA products were amplified using the anchor primer of dN6 and PrimeSTAR® Max DNA Polymerase (Takara, Dalian, China) on a Thermal Cycler (Bio-Rad, CA, USA). The product amplified by PCR using dN6 is not specific band, but a diffuse band. Second, the fragments from about 500 bp to about 1, 000 bp of the diffuse band were purified and ligated to the pClone007 using T-007Vsm Kit (Tsinke, Beijing, China) according to the instructions of the manufacturer and then transformed into competent cells of *Escherichia coli* DH5α. Fifty DH5α clones containing cDNA fragment of the dsRNA ranging from 500 to 1, 000 bp were sequenced in Tsingke Biotechnology company (Tsingke, Chongqing, China). The DNAMAN program and BLASTP program online version of the NCBI website were used to assemble and analyze these cloned sequences.

In order to obtain the 3′ and 5′ terminal sequence of the purified dsRNAs, a method described previously was carried out (Xie et al., 2006; Wang et al., 2013). Primers used for the 3′, 5′ RACE were listed in Table 1.

**Table 1:** Primers used in this manuscript.

	**Primer name**	**Nucleotide sequence**	**Position**
dsRNA2	Rs4bF1	AGTGTTTCGAACCGCATTGC	1117–1823 bp
	Rs4bR1	CGTCACCTGAGTTGTCGTCA	
	Rs4bF2	TCATTCAATGGCCCCTTCCC	427–1136 bp
	Rs4bR2	GCAATGCGGTTCGAAACACT	
dsRNA1	Rs4aF1	GCCACAGGATGCAAAAGCAA	380–1142 bp
	Rs4aR1	ACAATCATGCGTCGCACATG	
	Rs4aF2	ATGTGCGACGCATGATTGTG	1124–1881 bp
	Rs4aR2	ATGATGAACAGCGACCCCAG	
dsRNA2	6985-L1	TCAACAACTT TATCATGTTG TTCGATAGTG	Sequence-specific primers used to obtain the 5′- and 3′-terminal sequences of the dsRNAs
	6985-R1	CAAAGCACTG TTTTCATCCT TCCAACCATG	
dsRNA1	9860-L1	TCGAAATAGA TATTTCGGAT AGGGTATCGC	
	9860-R1	AACTTCCTGA GTTCGATATT CAGACCCCTG	

Multiple sequence alignments were performed on the whole amino acid sequences using MAFFT software (Rozewicki et al., 2019). Phylogenetic trees were constructed by using MEGA7 with maximum likelihood method (Kumar et al., 2016). Bootstrap values were evaluated with 1, 000 replicates. The sequences of partitiviruses used in this study were all downloaded from the NCBI database.

### Total RNA extraction and RT-PCR

Total RNAs were extracted with TRIzol following the manufacturer's instructions (Tiangen, Beijing, China). Four sets of primers were designed based on the nucleotide sequence of RsPV433 dsRNA1 and dsRNA2. Using 1, 000 ng total RNA extracted from the *R. solani* strains as template, reverse transcription was performed with 100 units of M-MLV reverse transcriptase and primed with 10 pM primer that complementary to the RsPV433 genome, the reactions were incubated at 42°C for 60 min followed by 75°C for 10 min to inactivate the reverse transcriptase. The PCR amplification was performed using the reverse transcribed cDNA as template. The primers used for the detection of RsPV433 are listed in Table 1.

### Biological testing

The colony diameters of each strain were measured daily for 4 d. The virulence of the TS10 and TS10-23 was determined according to a method previously described with slight modifications (Zhao et al., 2013). Pathogenicity tests were performed on 8-week-old seedlings of *Nicotiana tobacum* and *N. benthamiana*. Wounds were made by inserting a needle into every treated and control leaf. Leaves were either inoculated with 5 mm mycelial plugs from the edge of 4 d cultures of TS10 or TS10-23, or with PDA without mycelial plugs for the control. All the inoculated plants were incubated at 25°C for 2 days in a plastic container covered with an opaque cap to maintain high relative humidity and darkness. The cap was then removed and plants were grown under a 12:12 h (L:D) photoperiod for another 2 days. For the evaluation of the size of the *R. solani* caused lessions, we measured the longest and the shortest diameter of each lesion and then averaged them to get a radius. According to the formula for calculating the area of a circle, π is multiplied by the square of the radius, and finally the size of each lesion is obtained for statistical analysis.

### Statistical analysis

Data in this study were analyzed by SPSS soft-ware. One-way analysis of variance was used to compare the data, followed by Duncan's test at 5% level of significance to determine the significance of differences between treatment means.

## Results

### dsRNAs in *R. solani*

**Figure 1:**
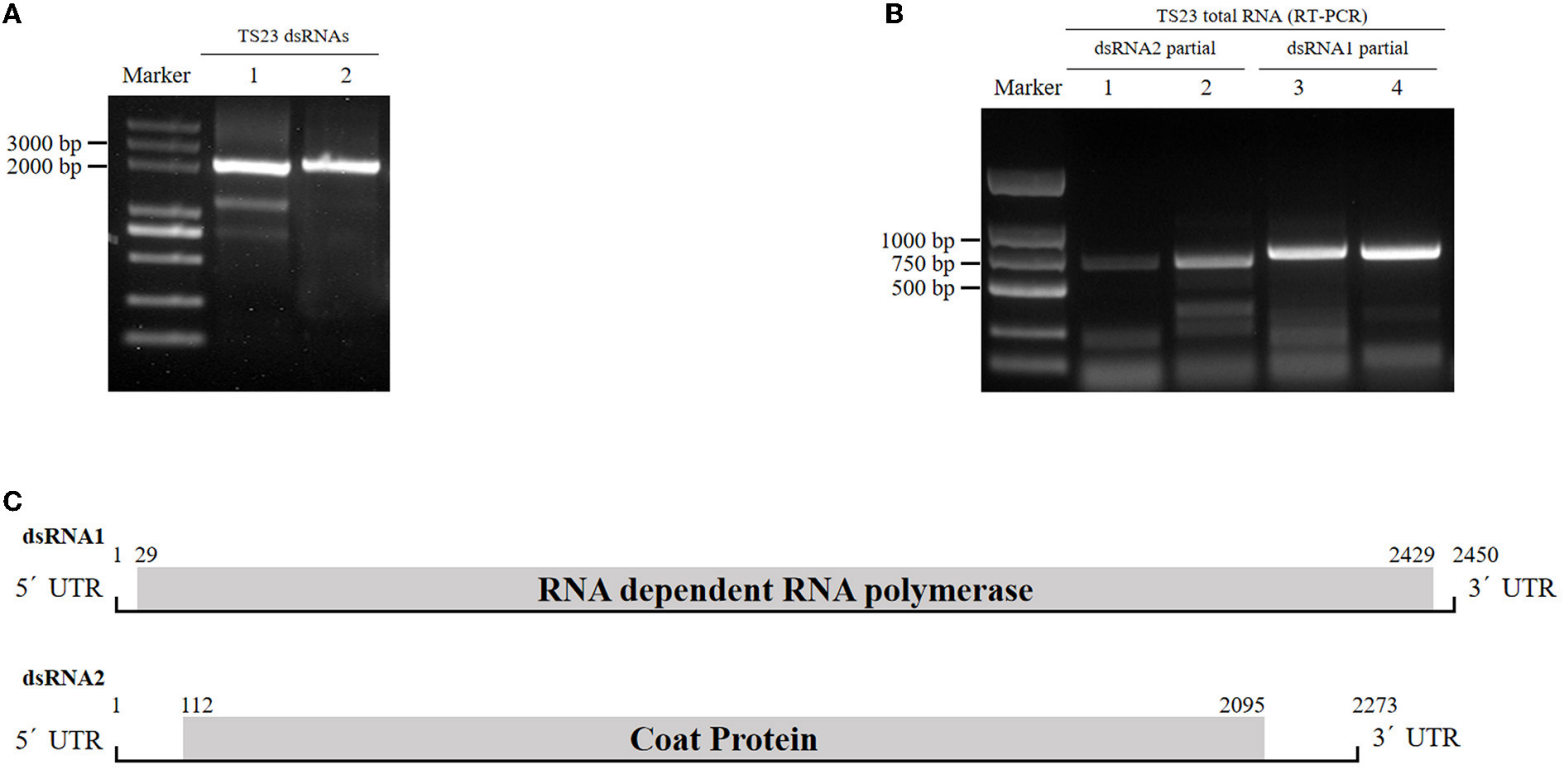
Identification of RsPV433 in *R. solani*. **(A)** Detection of dsRNA in mycelia of *R. solani* strain TS23. Lane 1 untreated dsRNA extracted from TS23, lane 2 DNase I and S1 nucleoase treated dsRNA extracted from TS23 strain. **(B)** Detection of RsPV433, using total RNA of TS23 and primers mentioned in the materials and methods section. **(C)** Schematic representation of the genome organization of RsPV433. RsPV433 contains two dsRNAs, and encode two proteins RdRp and CP, respectively. The ORF encoding RdRp and CP were marked in the figure.

When screened by a double-straned RNA extraction method, a *R. solani* strain TS23 was found to be dsRNA positive. After DNase I and S1 nuclease treated, a band with the size of about 2 kb still existed on the agarose gel (Figure 1A). Subsequent experiments revealed that the band actually comprised two dsRNA, which we named as dsRNA1 and dsRNA2.

### Nucleotide sequence and genome organization of RsPV433

By dsRNA cloning, the full-length cDNA sequences of dsRNA1 and dsRNA2 were obtained and separately submitted to GenBank (ID numbers ON665758 and ON665759). The lengths of dsRNA1 and dsRNA2 were 2450 and 2273 bp, respectively. dsRNA1 contains an open reading frame (ORF) predicted to encode the RNA-dependent RNA polymerase (RdRp), while dsRNA2 consisted of an ORF that might encode the coat protein (CP). Using BLASTP for the dsRNA1-encoded protein, we found that it was similar to the partitivirus-encoded RdRp, such as *Sarcosphaera coronaria* partitivirus (QLC36830.1, with 93% query cover and 60.85% identity), *Rosellinia necatrix* partitivirus 8 (YP_009449449.1, with 93% query cover and 62.13% identity), and *Fusarium poae* partitivirus 2 (YP_009272947.1, with 93% query cover and 60.51% identity). Thus, we tentatively named this novel virus Rhizoctonia solani prtitivirus 433 (RsPV433). The genome structure of RsPV433 is shown in Figure 1B. To detect the existence of RsPV433 in TS23, we designed four pairs of specific primers. We extracted the total RNA of TS23 and used it for RT-PCR amplification. The specific partial fragments of dsRNA1 and dsRNA2 with expected sizes were amplified from the total RNA of TS23, which confirmed the existence of RsPV433 in TS23 (Figure 1C).

### Phylogenetic analysis of RdRp and CP of RsPV433 in *Partitiviridae*

**Figure 2:**
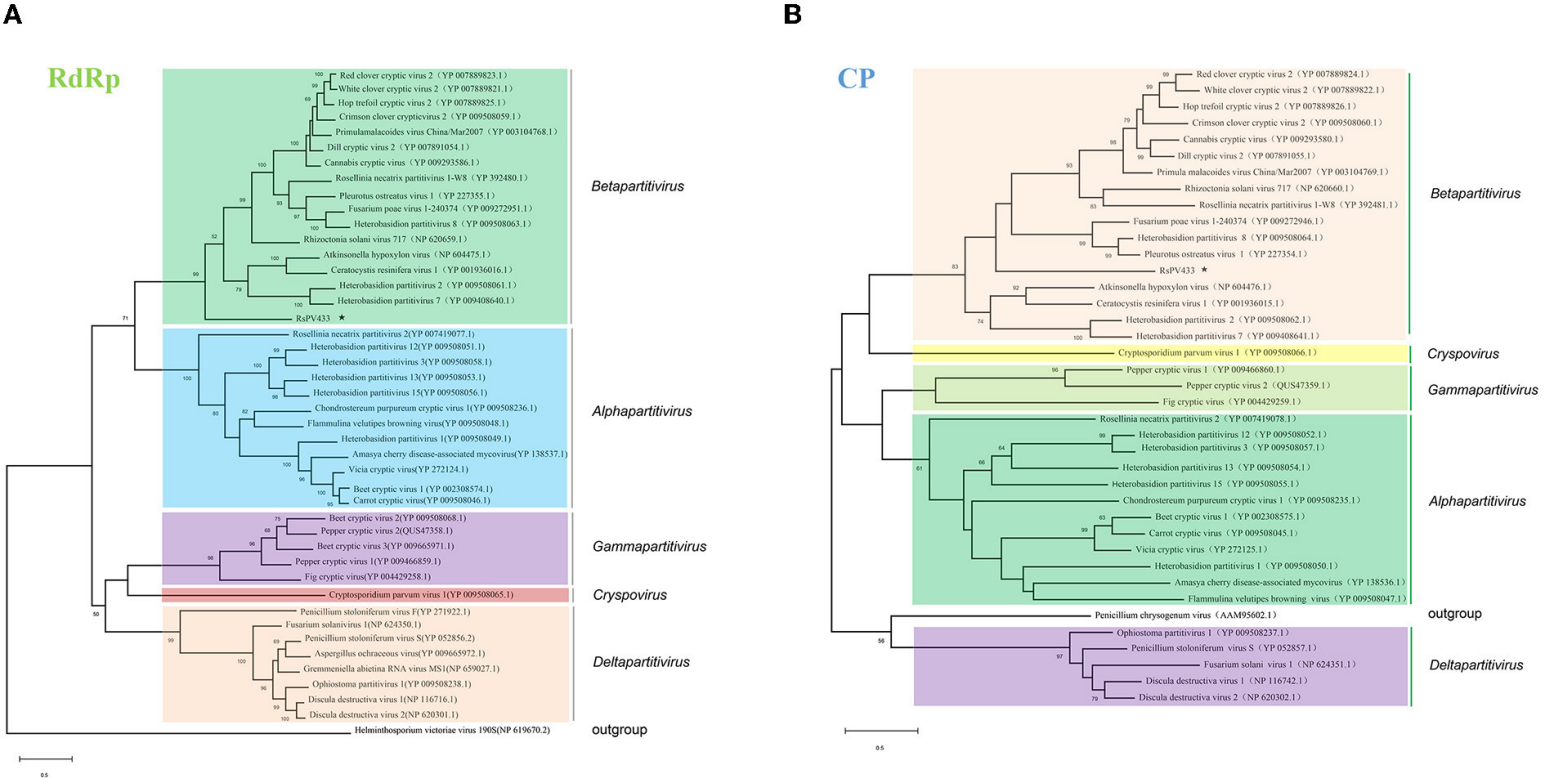
Phylogenetic analysis of RsPV433. Phylogenetic trees were generated based on the amino acid sequences of the RdRp and CP of RsPV433. **(A)** All viruses listed in NCBI virus taxonomy of *Partitiviridae* were chosed and their RdRp were selected and clustered in this phylogenetic tree, result suggested that RsPV433 is probably belongs to the *Betapartitivirius* of *Parititiviridae*. **(B)** Phylogenetic tree of viral coat protein encoded by partitiviruses. RsPV433 encoded coat protein was clustered in the *Betapartitivirus* clade.

The amino acid sequences of the RdRp of partitiviruses were used to build a phylogenetic tree through the maximum likelihood method to clarify the evaluation status of RsPV433. Results showed that RsPV433 was grouped in a clade of *Betapartitivirus* of the *Partitiviridae* (Figure 2A). To further validate this result, we performed a phylogenetic analysis of the CP amino acid sequence among partitiviruses (Figure 2B). Results also indicated that the RsPV433 clustered in a well-supported clade including *Rhizoctonia solani virus 717* and other members of the *Betapartitivirus*. Overall, based on phylogenetic analysis of RdRp and CP, our results suggest that RsPV433 might be a novel member of *Betapatitivirus* in *Partitiviridae*.

### Sequence similarity between RsPV433 and related species

**Figure 3:**
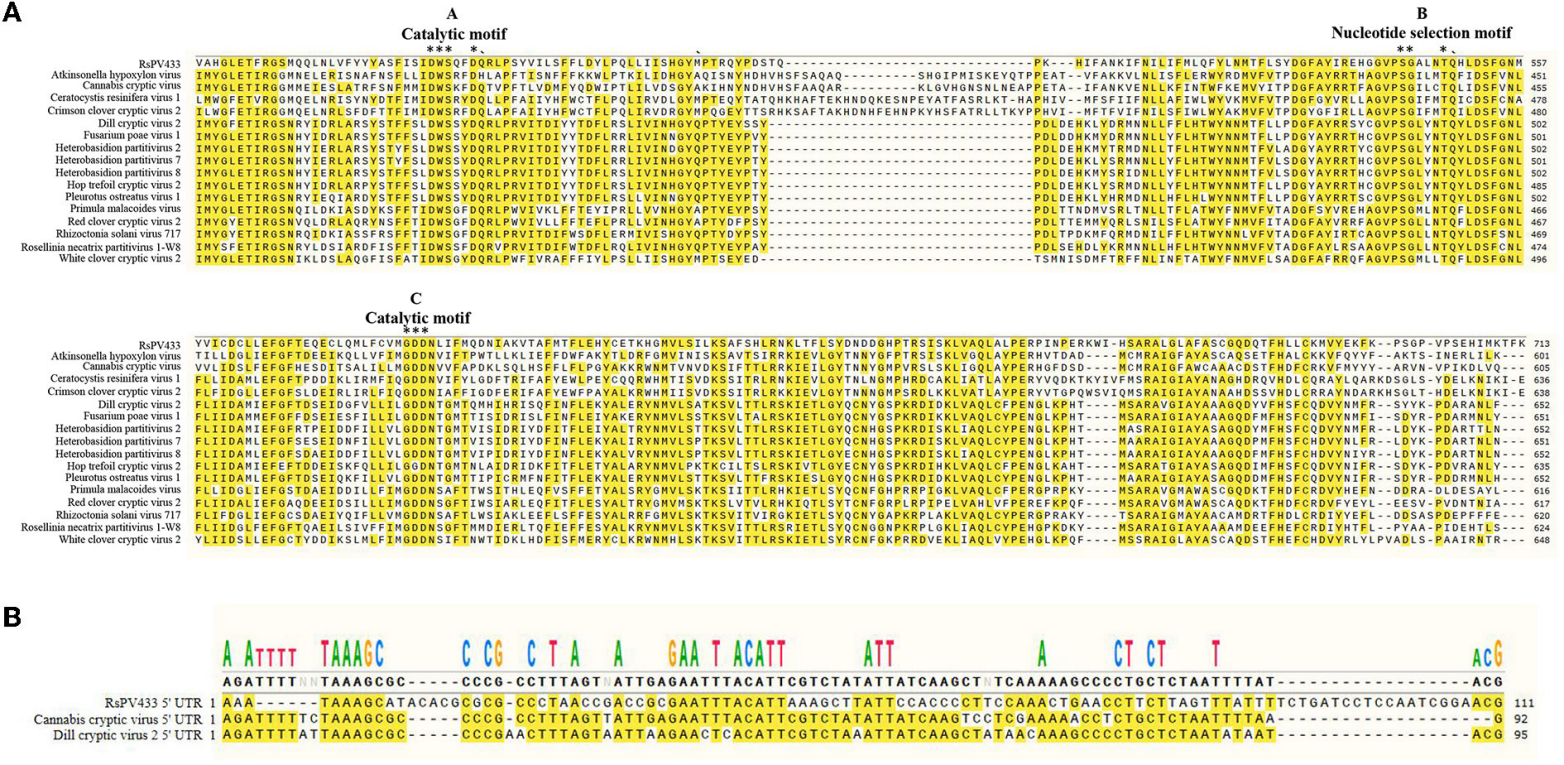
Sequence similarity analysises of RsPV433 and its relative species. **(A)** Multiple amino acid sequence alignment of RdRps of RsPV433 and other related mycoviruses in *Betapartitivirus*. The A-, B-, and C-motif were conserved motifs that exist in all RdRps of dsRNA viruses (Te Velthuis, 2014). **(B)** Nucleotide sequence alignment of the 5′ UTR of RsPV433 and two relative viruses (*Cannabis cryptic virus* and *Dill cryptic virus*).

Multiple sequence alignment analysis of RdRp encoded by RsPV433 with 15 members of the genus *Betapartitivirus* showed that RdRp of RsPV433 contains three conserved motifs (motifs A–C) that are well-conserved among dsRNA viruses (Figure 3A) (Te Velthuis, 2014). We also analyzed the similarity of the 5′ UTR region among the viruses that were close to RsPV433. The results showed that the 5′ UTR region of RsPV433 dsRNA2 was similar to its relatives (Figure 3B).

### Horizontal transmission of RsPV433

**Figure 4:**
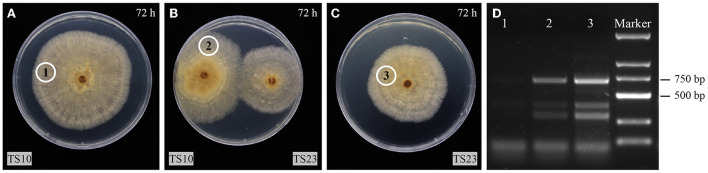
Horizontal transmission assay of RsPV433. **(A–C)** TS10 is a strain which is not infected by RsPV433, TS23 is a strain that is infected by RsPV433. All fungal were grow on PDA at 25°C in dark. The photos were taken at 72 h when the mycelial of TS10 and TS23 get in contact with each other. The cycled 1, 2, 3 implies the place where we take the mycelial plugs for RT-PCR detection. **(D)** RT-PCR detection of RsPV433, use the primer sets Rs4bF2/Rs4bR2 for the detection of RsPV433 dsRNA2. Lane 1, 2, 3 represent the sample we marked with cycle in **A–C**, samples were taken at 96 h when TS10 and TS23 have contacted for 24 h.

To investigate the horizontal transmission ability of RsPV433, TS10 and TS23 were inoculated on the same PDA plate. The strain TS23 served as a donor, while TS10, which was RsPV433-free, served as a recipient. After the mycelia of TS10 and TS23 were in contact for 24 h, samples (marked by circles in Figures 4A–C) were taken for RNA extraction. The total RNA of these samples were used for RT-PCR detection with the previously designed primer set (Rs4bF2/Rs4bR2) for detecting dsRNA2 of RsPV433. The RT-PCR result indicated that RsPV433 can be transmitted horizontally (Figure 4D).

### Effects of RsPV433 on *R. solani*

**Figure 5:**
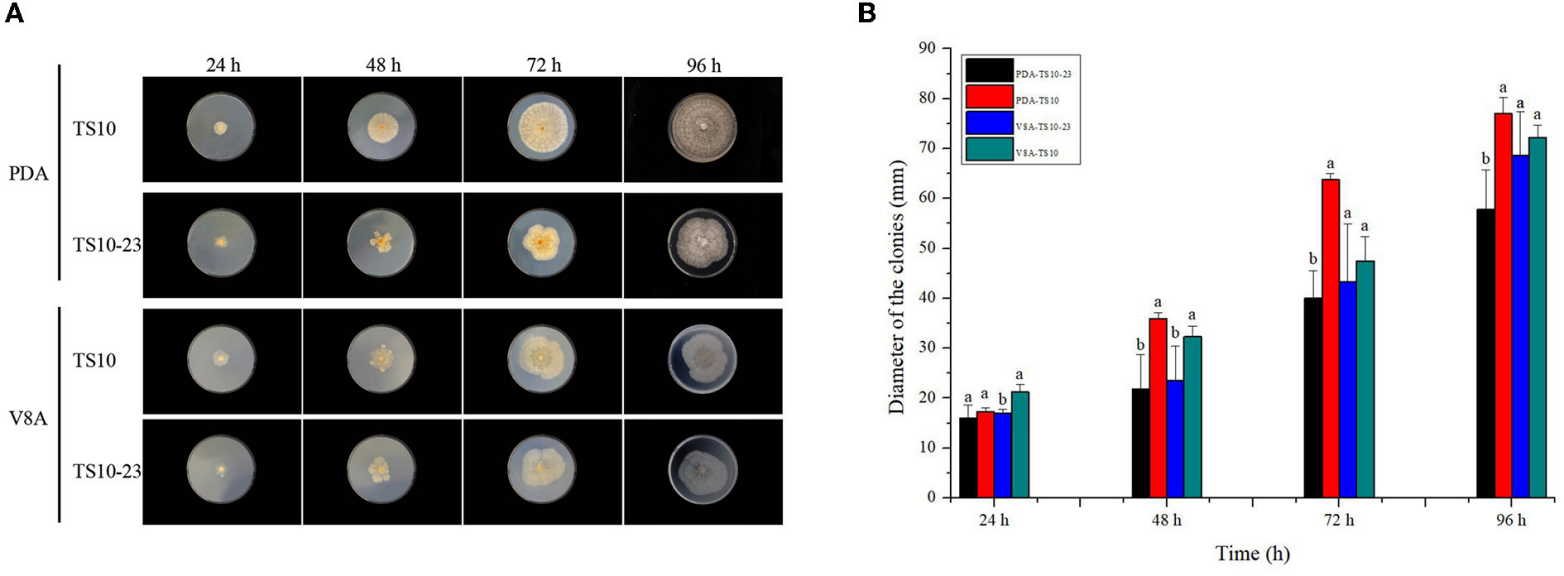
Growth phenotype of TS10 (RsPV433 free) and TS10-23 (RsPV433 infected) *R. solani* strain. **(A)** Growth phenotype of TS10 and TS10-23 on PDA and V8A medium at 25°C in dark for 4 days. **(B)** Comparison of the average radial mycelial growth rates between TS10 and TS10-23 on PDA and V8A, respectively.

**Figure 6:**
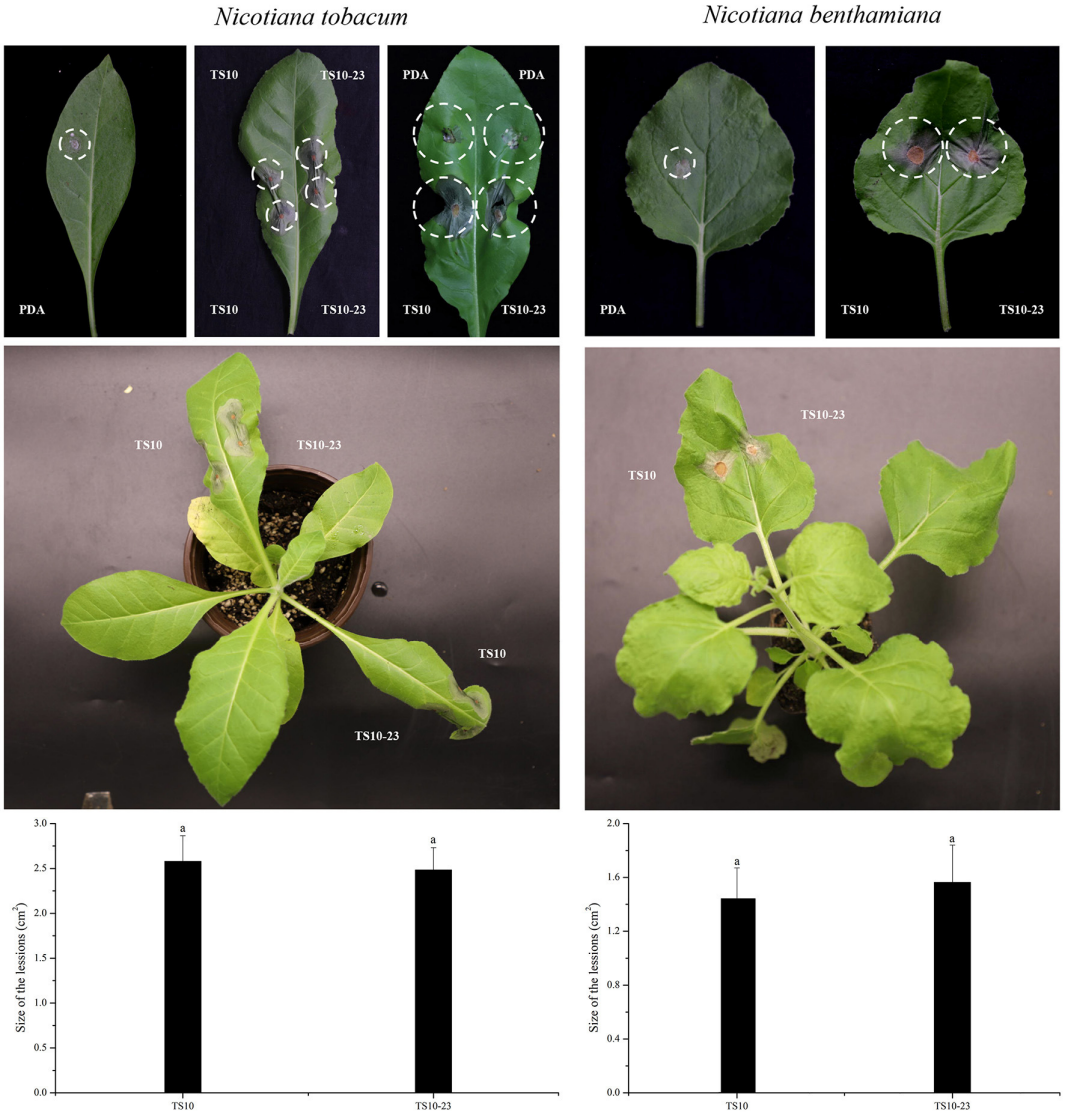
Pathogenicity test of *R. solani* that with and without RsPV433 on *N. tobacum* and *N. benthamiana*. The pathogenicity of TS10 and TS10-23 were tested by inoculating the mycelial plugs of these two strains on *N. tobacum* and *N. benthamiana*. Symptoms were observed 4 days after inoculation (2 days for dark treated and 2 days for natural day and night at 25°C), PDA refers to the mock treated. Result implied that the tested two strains have no significant difference in inducing lesions on *N. tobacum* and *N. benthamiana*.

To observe the effect of RsPV433 on the growth phenotype of *R. solani*, we inoculated TS10 and TS10-23 onto PDA medium and V8A medium, respectively. Results showed that *R. solani* infected by RsPV433 (TS10-23) grew much more slowly on PDA medium than *R. solani* that was RsPV433-free (TS10); however, TS10 and TS10-23 on the V8A medium showed almost the same growth pace (Figure 5). Then we tested the pathogenicity of TS10 and TS10-23 by inoculating these two strains on *N. tobacum* and *N. benthamiana*. Results showed that there was no significant difference between the area of lesions caused by TS10 and TS10-23 on *N. tobacum* and *N. benthamiana*, it implied that RsPV433 does not affect the lesion inducing ability of *R. solani* (Figure 6).

## Discussion

Due to the rapid development of high-throughput viromics, numerous new partitiviruses have been identified, which makes the taxonomy of partitiviruses chaotic. In addition to the five classical genera, some new clade that have not been officially admitted appeared, like the *Epsilonpartitivirus* and the *Zetapartitivirus* (Nerva et al., 2017; Gilbert et al., 2019; Jiang et al., 2019; Ahmed et al., 2020). In addition, when we analyzed the nucleotide sequences of viruses in the *Partitiviridae* family we found that the relative virus of dsRNA1 and dsRNA2 of some partitivirus is not the same virus, this enlarged the uncertainty of the classification of *Partitiviridae*. Our phylogenetic analysis results suggest that RsPV433 might be a new member of *Betapartitivirus* (Figure 2). However, Figure 2A shows that the gap between RsPV433 and its relative betapartitivirus is large. Furthermore, the sequence similarity between dsRNA1 and dsRNA2 of RsPV433 on the 5′ and 3′ terminal ends is not as high as we expected, the reason might be that the 5′ and 3′ terminal sequences of these two dsRNAs which we got is not long enough to cover the 5′ and 3′ terminal ends of RsPV433. Hence, the terminal sequences of RsPV433 might need to be further analyzed, the taxonomic status of RsPV433 could also need to be further elucidated in future study.

Vertical transmission through spores and horizontal transmission *via* hyphal anastomosis and heterokaryosis are the principle transmission pathways of mycoviruses (Brusini and Robin, 2013; Abdoulaye et al., 2019). Partitiviruses, for example, RsPV2, RsPV6, RsPV7, and RsPV8, can be transmitted vertically and horizontally (Jian et al., 1997; Chen et al., 2019). However, Liu et al. (2016) discovered that the SsHADV-1 can be transmitted extracellularly by the mycophagous insect vector *Lycoriella ingénue* beside the vertical and horizontal transmission pathway (Liu et al., 2016). In this study, we verified that RsPV433 can be transmitted horizontally to the *R. solani* strain within the same AG group, but it is unclear whether RsPV433 has other routes of transmission.

Mycovirus studies are often focused on viruses that cause hypovirulence to their host fungi, because some of them can be developed into biocontrol agents. The potential capability of mycovirus to be biocontrol agent was well-discussed in the published papers (Ghabrial and Suzuki, 2009; Xie and Jiang, 2014; García-Pedrajas et al., 2019; Rigling et al., 2020). For example, *Cryphonectria hypovirus 1* (CHV1), isolated from *Cryphonectria parasitica*, was successfully used to control chestnut blight in Europe, and Botryosphaeria dothidea partitivirus 2 (BdPV2) isolated from *Botryosphaeria dothidea* greatly reduced the virulence of its host and has great potential in controlling *B. dothidea* caused diseases (Feau et al., 2014; Wang et al., 2014). We found that RsPV433 has no significant impact on the lesion expansion of *R. solani* on *N. tobacum* and *N. benthamiana*. However, the size of lesion is only one aspect of evaluating the pathogenicity of fungal pathogens, more experiments are needed to verify the effect of RsPV433 on the pathogenicity of *R. solani*, because the ability of altering the host of its fungal host or helping other mycoviruses to alter its host could also affect the pathogenicity of mycovirus's host (Aihara et al., 2018; Tran et al., 2019).

The host phenotype on growth medium is a typical alternation mediated by mycoviruses on their hosts, and this implies that mycoviruses may directly interfere with the nutrient absorption and basic metabolism of its host (Kotta-Loizou, 2021). We speculate that the reason RsPV433-infected *R. solani* grew differently on PDA and V8A might be that in the growth phenotype investigation experiment V8A represented a complete medium, while PDA was equivalent to a deficient medium. Presence of RsPV433 interfered the ability of *R. solani* to overcome the nutrient deficiency and eventually cause TS10-23 to grow slower than TS10 on the PDA medium. This could help explain why there was no significant difference between TS10 and TS10-23 in inducing lesion on *N. tobacum* and *N. benthamiana*, because both *N. tobacum* and *N. benthamiana* that we used in the pathogenicity test may represent a complete medium like V8A did (Figure 5). Nevertheless, the mechanism of how RsPV433 affects the growth of *R. solani* on the PDA medium needs further research.

## Data availability statement

The datasets presented in this study can be found in online repositories. The names of the repository/repositories and accession number(s) can be found below: GenBank and the accession numbers are ON665758 and ON665759.

## Author contributions

XC and NJ designed the research. XC, NJ, ZY, and YS performed all experiments. XC and MY analyzed data. XC and NJ wrote the manuscript. All authors reviewed the final manuscript. All authors contributed to the article and approved the submitted version.

## Funding

This study received funding from China National Science Foundation (Grant No. 32001870), Guizhou provincial Science and Technology projects [ZK(2021) normal 136]. Tobacco Pests and Diseases Green Prevention and Control Major Special Project [110202101045(LS-05)] and Project of Yunnan Company of China Tobacco Corporation (2021530000242031). The funder was not involved in the study design, collection, analysis, interpretation of data, the writing of this article or the decision to submit it for publication.

## Conflict of interest

The authors declare that the research was conducted in the absence of any commercial or financial relationships that could be construed as a potential conflict of interest.

## Publisher's note

All claims expressed in this article are solely those of the authors and do not necessarily represent those of their affiliated organizations, or those of the publisher, the editors and the reviewers. Any product that may be evaluated in this article, or claim that may be made by its manufacturer, is not guaranteed or endorsed by the publisher.

## References

[B1] Abdoulaye A. H. Foda M. F. Kotta-Loizou I. (2019). Viruses infecting the plant pathogenic fungus *Rhizoctonia solani*. Viruses 11:1113. 10.3390/v11121113 31801308 PMC6950361

[B2] Ahmed I. Li P. F. Zhang L. H. Jiang X. W. Bhattacharjee P. Guo L. H.. (2020). First report of a novel partitivirus from the phytopathogenic fungus *Fusarium cerealis* in China. Arch. Virol. 165, 2979–2983. 10.1007/s00705-020-04802-4 32902666

[B3] Aihara M. Urayama S. I. Le M. T. Katoh Y. Higashiura T. Fukuhara T.. (2018). Infection by Magnaporthe oryzae chrysovirus 1 strain a triggers reduced virulence and pathogenic race conversion of its host fungus, *Magnaporthe oryzae*. J. Gen. Plant Pathol. 63, 10–18. 10.1007/s10327-018-0766-7

[B4] Bian R. Andika I. B. Pang T. Lian Z. Wei S. Niu E.. (2020). Facilitative and synergistic interactions between fungal and plant viruses. Proc. Natl. Acad. Sci. U. S. A. 117, 3779–3788. 10.1073/pnas.1915996117 32015104 PMC7035501

[B5] Brusini J. Robin C. (2013). Mycovirus transmission revisited by *in situ* pairings of vegetatively incompatible isolates of *Cryphonectria parasitica*. J. Virol. Methods 187, 435–442. 10.1016/j.jviromet.2012.11.025 23201291

[B6] Chen Y. Gai T. X. Chen R. X. Li C. X. Zhao G. K. Xia Y. Z.. (2019). Characterization of three novel betapartitiviruses co-infecting the phytopathogenic fungus *Rhizoctonia solani*. Virus Res. 270:197649. 10.1016/j.virusres.2019.197649 31276695

[B7] Feau N. Dutech C. Brusini J. Rigling D. Robin C. (2014). Multiple introductions and recombination in Cryphonectria hypovirus 1: perspective for a sustainable biological control of chestnut blight. Evol. Appl. 7, 580–596. 10.1111/eva.12157 24944571 PMC4055179

[B8] Feng W. Hieno A. Otsubo K. Suga H. Kageyama K. (2022). Emergence of self-fertile *Phytophthora colocasiae* is a possible reason for the widespread expansion and persistence of taro leaf blight in Japan. Mycol. Prog. 2022, 21, 49–58. 10.1007/s11557-021-01762-0

[B9] García V. G. Onco M. P. Susan V. R. (2006). Biology and systematics of the form genus *Rhizoctonia*. Span. J. Agric. Res. 4, 55–79. 10.5424/sjar/2006041-178

[B10] García-Pedrajas M. D. Cañizares M. C. Sarmiento-Villamil J. L. Jacquat A. G. Dambolena J. S. (2019). Mycoviruses in biological control: from basic research to field implementation. Phytopathology 109, 1828–1839. 10.1094/PHYTO-05-19-0166-RVW 31398087

[B11] Ghabrial S. A. Castón J. R. Jiang D. H. Nibert M. L. Suzuki N. (2015). 50-plus years of fungal viruses. Virology 479, 356–368. 10.1016/j.virol.2015.02.034 25771805

[B12] Ghabrial S. A. Suzuki N. (2009). Viruses of plant pathogenic fungi. Annu. Rev. Phytopathol. 47, 353–384. 10.1146/annurev-phyto-080508-081932 19400634

[B13] Gilbert K. B. Holcomb E. E. Allscheid R. L. Carrington J. C. (2019). Hiding in plain sight: new virus genomes discovered *via* a systematic analysis of fungal public transcriptomes. PLoS ONE 14:e0219207. 10.1371/journal.pone.0219207 31339899 PMC6655640

[B14] Gonzalez M. Pujol M. Metraux J. P. Gonzalez-Garcia V. Borrás-Hidalgo O. (2011). Tobacco leaf spot and root rot caused by *Rhizoctonia solani* kühn. Mol. Plant Pathol. 12, 209–216. 10.1111/j.1364-3703.2010.00664.x 21355993 PMC6640363

[B15] Hillman B. I. Annisa A. Suzuki N. (2017). Viruses of plant-interacting fungi. Adv. Virus Res. 100, 99–116. 10.1016/bs.aivir.2017.10.003 29551145

[B16] Jian J. H. Lakshman D. K. Tavantzis Stellos M. (1997). Association of distinct double-stranded RNAs with enhanced or diminished virulence in *Rhizoctonia solani* infecting potato. Mol. Plant Microbe Interact. 10, 1002–1009. 10.1094/MPMI.1997.10.8.100218471880

[B17] Jiang Y. H. Wang J. X. Yang B. Wang Q. R. Zhou J. J. Yu W. F. (2019). Molecular characterization of a debilitation-associated partitivirus infecting the pathogenic fungus *Aspergillus flavus*. Front. Microbiol. 10:626. 10.3389/fmicb.2019.00626 30984147 PMC6447663

[B18] Kamitani M. Okuno T. Kudoh H. (2020). Complete genome sequence of a novel partitivirus from a wild brassicaceous plant, *Arabidopsis halleri*. Arch. Virol. 165, 2091–2094. 10.1007/s00705-020-04670-y 32533330

[B19] Kondo H. Botella L. Suzuki N. (2022). Mycovirus diversity and evolution revealed/inferred from recent studies. Annu. Rev. Phytopathol. 60, 30. 10.1146/annurev-phyto-021621-122122 35609970

[B20] Kondo H. Chiba S. Toyoda K. Suzuki N. (2013). Evidence for negative-strand RNA virus infection in fungi. Virology 435, 201–209. 10.1016/j.virol.2012.10.002 23099204

[B21] Kotta-Loizou I. (2021). Mycoviruses and their role in fungal pathogenesis. Curr. Opin. Microbiol. 63, 10–18. 10.1016/j.mib.2021.05.007 34102567

[B22] Kumar S. Stecher G. Tamura K. (2016). MEGA7: molecular evolutionary genetics analysis Version 7.0 for bigger datasets. Mol. Biol. Evol. 33, 1870–1874. 10.1093/molbev/msw054 27004904 PMC8210823

[B23] Li P. F. Zhang H. L. Chen X. G. Qiu D. W. Guo L. H. (2015). Molecular characterization of a novel hypovirus from the plant pathogenic fungus *Fusarium graminearum*. Virology 481, 151–160. 10.1016/j.virol.2015.02.047 25781585

[B24] Li Y. T. Li S. W. Zhao Y. M. Zhou T. Wu X. H. Zhao C. (2022b). Six novel mycoviruses containing positive single-stranded RNA and double-stranded RNA genomes co-infect a single strain of the *Rhizoctonia solani* AG-3 PT. Viruses 14:813. 10.3390/v14040813 35458543 PMC9025235

[B25] Liu S. Xie J. T. Cheng J. S. Li B. Chen T. Fu Y. P.. (2016). Fungal DNA virus infects a mycophagous insect and utilizes it as a transmission vector. Proc. Natl. Acad. Sci. U. S. A. 113, 12803–12808. 10.1073/pnas.1608013113 27791095 PMC5111676

[B26] MatRazali N. Hisham S. N. Kumar I. S. Shukla R. N. Lee M. Abu Bakar M. F.. (2021). Comparative genomics: insights on the pathogenicity and lifestyle of *Rhizoctonia solani*. Int. J. Mol. Sci. 22:2183. 10.3390/ijms22042183 33671736 PMC7926851

[B27] Mercado Cárdenas G. Galván M. Barrera V. Carmona M. (2012). First report of target spot of tobacco caused by *Rhizoctonia solani* AG-2.1. Plant Dis. 96:456. 10.1094/PDIS-08-11-0696 30727115

[B28] Morris T. J. Dodds J. A. (1979). Isolation and analysis of double-stranded RNA from virusinfected plant and fungal tissue. Phytopathology 69, 854–858. 10.1094/Phyto-69-854 40934407

[B29] Myers J. M. James T. Y. (2022). Mycoviruses. Curr. Biol. 32, 150–155. 10.1016/j.cub.2022.01.049 35231405

[B30] Nerva L. Silvestri A. Ciuffo M. Palmano S. Varese G. C. Turina M. (2017). Transmission of Penicillium aurantiogriseum partiti-like virus 1 to a new fungal host (*Cryphonectria parasitica*) confers higher resistance to salinity and reveals adaptive genomic changes. Environ. Microbiol. 19, 4480–4492. 10.1111/1462-2920.13894 28836717

[B31] Nibert M. L. Ghabrial S. A. Maiss E. Lesker T. Vainio E. J. Jiang D.. (2014). Taxonomic reorganization of family *Partitiviridae* and other recent progress in partitivirus research. Virus Res. 188, 128–141. 10.1016/j.virusres.2014.04.007 24768846

[B32] Nibert M. L. Woods K. M. Upton S. J. Ghabrial S. A. (2009). *Cryspovirus*: a new genus of protozoan viruses in the family *Partitiviridae*. Arch. Virol. 154, 1959–1965. 10.1007/s00705-009-0513-7 19856142

[B33] Petrzik K. (2019). Evolutionary forces at work in partitiviruses. Virus Genes 55, 563–573. 10.1007/s11262-019-01680-0 31230256

[B34] Rigling D. Robin C. Prospero S. (2020). Mycovirus-mediated biological control. Encyclopedia of Virology 4, 468–477 10.1016/B978-0-12-809633-8.21516-1

[B35] Rozewicki J. Li S. L. Amada K. M. Standley D. M. Katoh K. (2019). MAFFT-DASH: integrated protein sequence and structural alignment. Nucleic Acids Res. 47, W5–W10. 10.1093/nar/gkz342 31062021 PMC6602451

[B36] Ruiz-Padilla A. Rodríguez-Romero J. Gómez-Cid I. Pacifico D. Ayllón M. A. (2021). Novel mycoviruses discovered in the mycovirome of a necrotrophic fungus. MBio 12, e03705–e03720. 10.1128/mBio.03705-20 33975945 PMC8262958

[B37] Shah U. A. Kotta-Loizou I. Fitt B. D. L. Coutts R. H. A. (2020). Mycovirus-induced hypervirulence of *Leptosphaeria biglobosa* enhances systemic acquired resistance to *Leptosphaeria maculans* in *Brassica napus*. Mol. Plant Microbe Interact. 33, 98–107. 10.1094/MPMI-09-19-0254-R 31652089

[B38] Shew H. D. (1991). Infection and development of target spot of flue-cured tobacco caused by *Thanatephorus cucumeris*. Plant Dis. 74, 1009–1013. 10.1094/PD-74-1009

[B39] Shiba K. Hatta C. Sasai S. Tojo M. Ohki S. T. Mochizuki T. (2018). Genome sequence of a novel partitivirus identified from the oomycete *Pythium nunn*. Arch. Virol. 163, 2561–2563. 10.1007/s00705-018-3880-0 29754306

[B40] Sun M. Shi C. H. Ju L. Wang H. C. Cai L. Liu T.. (2022). First report of target spot caused by *Rhizoctonia solani* AG-6 in tobacco in China. Plant Dis. 10.1094/PDIS-09-21-2077-PDN35253480

[B41] Tang L. G. Song L. P. Lin C. F. Wang B. C. Lin J. Z. Gao C. B.. (2021). Complete nucleotide sequence of a novel partitivirus from *Brassica campestris* L. ssp. chinensis. Arch. Virol. 166, 1775–1778. 10.1007/s00705-021-05041-x 33772366

[B42] Te Velthuis A. J. (2014). Common and unique features of viral RNA-dependent polymerases. Cell Mol. Life Sci. 71, 4403–4420. 10.1007/s00018-014-1695-z 25080879 PMC4207942

[B43] Tran T. T. Li H. Nguyen D. Q. Jones M. G. K. Wylie S. J. (2019). Co-infection with three mycoviruses stimulates growth of a *Monilinia fructicola* isolate on nutrient medium, but does not induce hypervirulence in a natural host. Viruses. 11:89. 10.3390/v11010089 30669656 PMC6356717

[B44] Vainio E. J. Chiba S. Ghabrial S. A. Maiss E. Roossinck M. Sabanadzovic S.. (2018). ICTV virus taxonomy profile: *Partitiviridae*. J. Gen. Virol. 99, 17–18. 10.1099/jgv.0.000985 29214972 PMC5882087

[B45] Wang L. P. Jiang J. J. Wang Y. F. Hong N. Zhang F. P. Xu W. X.. (2014). Hypovirulence of the phytopathogenic fungus *Botryosphaeria dothidea*: association with a coinfecting chrysovirus and a partitivirus. J. Virol. 88, 7517–7527. 10.1128/JVI.00538-14 24760881 PMC4054428

[B46] Wang S. C. Ahmed I. Li X. H. Nie J. H. Guo L. H. (2022a). Evidence for a novel partitivirus isolated from the entomopathogenic nematode *Steinernema ceratophorum*. Arch. Virol. 167, 969–972. 10.1007/s00705-021-05314-5 35112200

[B47] Wang S. C. Kondo H. Liu L. Guo L. H. Qiu D. W. (2013). A novel virus in the family *Hypoviridae* from the plant pathogenic fungus *Fusarium graminearum*. Virus Res. 174, 69–77. 10.1016/j.virusres.2013.03.002 23499998

[B48] Wang X. Y. Lai J. L. Hu H. H. Yang J. R. Zang K. Zhao F. Y.. (2022b). Infection of Nigrospora nonsegmented RNA virus 1 has important biological impacts on a fungal host. Viruses 14:795. 10.3390/v14040795 35458525 PMC9029208

[B49] Wang Y. F. Zhao H. Cao J. Y. Yin X. M. Guo Y. S. Guo L. H.. (2022c). Characterization of a novel mycovirus from the phytopathogenic fungus *Botryosphaeria dothidea*. Viruses 14:331. 10.3390/v14020331 35215923 PMC8879742

[B50] White T. J. Bruns S. Lee S. Taylor J. (1990). Amplification and direct sequencing of fungal ribosomal RNA genes for phylogenetics. PCR Protocols 1, 315–322. 10.1016/B978-0-12-372180-8.50042-1

[B51] Wu Y. H. Zhao Y. Q. Fu Y. Zhao X. X. Chen J. G. (2012). First report of target spot of flue-cured tobacco caused by *Rhizoctonia solani* AG-3 in China. Plant Dis. 96:1824. 10.1094/PDIS-06-12-0551-PDN 30727290

[B52] Xie J. Wei D. M. Jiang D. H. Fu Y. P. Li G. Q. Ghabrial S.. (2006). Characterization of debilitation-associated mycovirus infecting the plant-pathogenic fungus *Sclerotinia sclerotiorum*. J. Gen. Virol. 87, 241–249. 10.1099/vir.0.81522-0 16361437

[B53] Xie J. T. Jiang D. H. (2014). New insights into mycoviruses and exploration for the biological control of crop fungal diseases. Annu. Rev. Phytopathol. 52, 45–68. 10.1146/annurev-phyto-102313-050222 25001452

[B54] Xie J. T. Xiao X. Q. Fu Y. P. Liu H. Q. Cheng J. S. Ghabrial S. A.. (2011). A novel mycovirus closely related to hypoviruses that infects the plant pathogenic fungus *Sclerotinia sclerotiorum*. Virology 418, 49–56. 10.1016/j.virol.2011.07.008 21813149

[B55] Xu G. Zhang X. C. Liang X. F. Chen D. P. Xie C. P. Kang Z. S.. (2022). A novel hexa-segmented dsRNA mycovirus confers hypovirulence in the phytopathogenic fungus *Diaporthe pseudophoenicicola*. Environ. Microbiol. 10.1111/1462-2920.15963 35315558

[B56] Xu M. L. Hao K. Q. Yang J. G. Wang F. L. Xiao Z. X. Li W. (2018). First report of *Rhizoctonia solani* AG-3 causing tobacco target spot in Yunnan, China. Plant Dis. 10.1094/PDIS-02-18-0249-PDN30113255

[B57] Yang Y. G. Zhao C. Guo Z. J. Wu X. H. (2015). Anastomosis group and pathogenicity of *Rhizoctonia solani* associated with stem canker and black scurf of potato in China. Eur. J. Plant Pathol. 143, 99–111. 10.1007/s10658-015-0668-x

[B58] Zhai L. F. Xiang J. Zhang M. X. Fu M. Yang Z. K. Hong N.. (2016). Characterization of a novel double-stranded RNA mycovirus conferring hypovirulence from the phytopathogenic fungus *Botryosphaeria dothidea*. Virology 493, 75–85. 10.1016/j.virol.2016.03.012 27015523

[B59] Zhang H. X. Xie J. T. Fu Y. P. Cheng J. S. Qu Z. Zhao Z. Z.. (2020). A 2-kb Mycovirus converts a pathogenic fungus into a beneficial endophyte for *Brassica* protection and yield enhancement. Mol. Plant 13, 1420–1433. 10.1016/j.molp.2020.08.016 32998002

[B60] Zhao Y. Q. Wu Y. H. Fu Y. Chen J. G. An M. N. Zhao X. X. (2013). Characterization of *Rhizoctonia solani* AG-3 isolates causing target spot of flue-cured tobacco in China. Adv. Materials Res. 726, 4321–4325. 10.4028/www.scientific.net/AMR.726-731.4321

[B61] Zhong J. Yang Z. Z. Yang X. Guo Z. J. Xie W. Zhang Y. J. (2021). Molecular characterization of a novel partitivirus and a fusarivirus coinfecting the fungus *Nigrospora sphaerica*. Arch. Virol. 166, 2325–2331. 10.1007/s00705-021-05095-x 34057607

